# Neuropsychiatric Assessment Before and After Epilepsy Surgery: A Descriptive Study in Patients of the National Institute of Neurology and Neurosurgery Manuel Velasco Suárez

**DOI:** 10.7759/cureus.81213

**Published:** 2025-03-26

**Authors:** David Daniel González Mille, Fernando Moisés Chávez Hassan, Daniel Crail-Meléndez, Ángel Alberto Ruiz Chow, Iris E. Martínez-Juárez

**Affiliations:** 1 Psychiatry Residency Program, National Institute of Neurology and Neurosurgery Manuel Velasco Suárez, Mexico City, MEX; 2 Neurology Department, Hospital Bautista de Nicaragua, Managua, NIC; 3 Neuropsychiatry Department, National Institute of Neurology and Neurosurgery Manuel Velasco Suárez, Mexico City, MEX; 4 Epilepsy Clinic, National Institute of Neurology and Neurosurgery Manuel Velasco Suárez, Mexico City, MEX

**Keywords:** epilepsy, epilepsy surgery, neuropsychiatry, neurosurgery, psychiatric sequelae

## Abstract

Background: The relationship between epilepsy and psychiatric disorders is complex and is influenced by biological and psychological factors and social determinants. There is evidence that psychiatric disorders are more common in patients with epilepsy compared to the general population. The impact of epilepsy surgery on psychiatric comorbidity is less clear, and patients who are candidates for epilepsy surgery should undergo several investigations, including a longitudinal psychiatric diagnosis before and after as part of the evaluation of the potential success of the surgery.

Objective: The objective of the study is to determine the prevalence of psychiatric disorders in patients undergoing epilepsy surgery before and after the surgical intervention.

Methodology: We analyzed the data of 95 patients who underwent epilepsy surgery at the National Institute of Neurology and Neurosurgery (Mexico) between 2006 and 2012 and who were assessed by psychiatry before and after the surgery.

Results: There were no significant differences in the prevalence of psychiatric disorders before and after epilepsy surgery (odds ratio (OR): 1.371 (0.760-2.473), p = 0.294).

Conclusion: Our results are consistent with those previously reported in the medical literature. Epilepsy surgery is a procedure that requires a multidisciplinary approach, and all patients undergoing the procedure should receive a longitudinal follow-up by psychiatry.

## Introduction

Neuropsychiatric symptoms in epilepsy

There are a large number of studies that conclude that psychiatric disorders are more frequent in patients with epilepsy, compared to the general population. For example, one study concluded that people with epilepsy have a two to five times higher risk of developing any psychiatric disorder and that one in three patients with epilepsy have a psychiatric diagnosis in their lifetime [1].

The relationship between epilepsy and psychiatric disorders is complex and is influenced by factors such as the age of onset, duration of illness, interictal epileptiform discharges, seizure frequency, and side effects of antiseizure medication [2]. However, a wide range of psychosocial factors affecting people living with epilepsy should also be considered, e.g., restricted job opportunities, lower income, lower chances of finding a partner, discrimination, and stigmatization [3]. Also, several studies have indicated a bidirectional relationship between epilepsy and psychiatric disorders, meaning that patients with epilepsy have an increased risk of developing mental disorders, and patients with mental disorders have an increased risk of developing epilepsy, which has been demonstrated in psychotic disorders, depression, suicidal behavior, attention-deficit/hyperactivity disorder (ADHD), and autism spectrum disorder [4].

Epilepsy surgery and psychiatric complications

It is considered that 60%-70% of patients with focal epilepsy are seizure-free with medication; however, 30%-40% of the remaining patients continue to have seizures and may fall within the definition of refractory epilepsy, which is one in which treatment with two or three anticrisis drugs has been ineffective [5]. Epilepsy surgery is now widely accepted as an option for the treatment of epilepsy, which should be considered as a possibility in all patients who meet the definition of refractoriness and is linked not only to better seizure control but also to an improvement in the quality of life in several spheres [5].

Patients who are candidates for epilepsy surgery should undergo a thorough evaluation, which aims to locate the epileptogenic zone and its relationship with eloquent areas of the brain, but without forgetting the other dimensions of epilepsy as a disease, for example, psychosocial comorbidity. The evaluation should always be conducted by an epilepsy specialist and should include at least magnetic resonance imaging, electroencephalogram, neuropsychological tests, and presurgical evaluation by a specialist in psychiatry, considering that up to 25%-40% of patients proceed to surgery with these studies; however, others will require more in-depth investigations, such as nuclear medicine studies and invasive techniques, such as intracranial electroencephalogram, in 10%-30% of patients [5].

## Materials and methods

Method

This study was approved by the Research Committee and Ethics Committee of the National Institute of Neurology and Neurosurgery Manuel Velasco Suárez, reference number 083/2024. The information was obtained from the database of the Epilepsy Clinic at the institute and the medical records. Data from patients who attended the clinic between 2006 and 2012 were analyzed. Since this is an observational study, from which data were collected from the medical record, a waiver of informed consent was requested and approved.

Participants

Participants were recruited from the Outpatient Clinic at the institute. The inclusion criteria were age (minimum 18 years) and patients who attended the Epilepsy Clinic at the National Institute of Neurology and Neurosurgery (Mexico) between 2006 and 2012, underwent epilepsy surgery, had follow-up for at least six months, and underwent psychiatric evaluation and neuropsychological tests before and after the surgery. From 2006 to 2012, 204 patients were presented to the Epilepsy Committee; of these, 165 patients with available and complete records were analyzed. Of the 165 patients, 95 underwent surgery; the remaining patients were not candidates for surgery, refused surgery, or were on the waiting list. Selected demographic and clinical characteristics are shown in Table 1.

**Table 1:** Descriptive statistics M: mean; SD: standard deviation; ACM: antiseizure medication

	N	Minimum	Maximum	M	SD
Current age	95	18	60	35.54	9.765
Age of onset of seizures	95	10	30.00	9.3389	7.28933
Age at the time of surgery	94	12.00	57.00	32.4574	9.95005
ACM before surgery	95	1	8	3.22	1.213
ACM after surgery	92	0	5	2.45	1.142

Measure

Psychiatric diagnoses and follow-ups were made by the psychiatry team of the institute, based on the criteria of the Diagnostic and Statistical Manual of Mental Disorders, Fourth Edition (DSM-IV), which was in effect at that time, according to our methodological design.

Statistical analyses

The SPSS program version 24 (IBM Corp., Armonk, NY, US) was used to analyze data. As this is a descriptive study, central tendency measures were used for numerical variables and percentages for nominal variables. A comparative analysis was performed to determine the odds ratio (OR), comparing the frequency of neuropsychiatric symptoms before and after surgery.

## Results

The diagnoses of patients who underwent surgery correspond to temporal lobe epilepsy (n = 70/73.7%), extratemporal epilepsy (n = 10/10.5%), Lennox-Gastaut Syndrome (n = 13/13.7%), and others (n = 2/2.1%) (Table 2).

**Table 2:** Diagnosis and type of epilepsy of the patients included in this study

Diagnosis	n	%
Right neocortical temporal lobe epilepsy	20	21.1
Left amygdalohippocampal temporal lobe epilepsy	19	20
Left neocortical temporal lobe epilepsy	18	18.9
Right amygdalohippocampal temporal lobe epilepsy	13	13.7
Lennox-Gastaut Syndrome	13	13.7
Lesional extratemporal epilepsy	8	8.4
Non-lesional extratemporal epilepsy	2	2.1
Others	2	2.1
Total	95	100

After surgery, 66.3% (n = 63) of patients presented with psychiatric illness, with the following diagnoses: depression 24.2% (n = 23), intellectual disability 14.7% (n = 14), cognitive impairment 7.4% (n = 7), anxiety 6.3% (n = 6), interictal psychosis 5.3% (n = 5), irritability 4.2% (n = 4), conversion disorder 2.1% (n = 2), and apathy 2.1% (n = 2). Nineteen (30.1%) patients had a diagnosis of more than one psychiatric disorder: cognitive impairment in five, anxiety in four, depression in four, irritable mood in three, conversion disorder in two, and apathy in one (Table 3).

**Table 3:** Psychiatric disorders before and after surgery

	Before surgery		After surgery		p
	Frequency	Percentage	Frequency	Percentage	
No disorder	39	41.1	32	33.7	0.5251
Depression	17	17.9	23	24.2	0.6360
Intellectual disability	14	14.7	14	14.7	1.0000
Anxiety	10	10.5	6	6.3	0.7824
Cognitive impairment	5	5.3	7	7.4	0.8894
Irritable mood	4	4.2	4	4.2	1.0000
Interictal psychosis	3	3.2	5	5.3	0.8969
Conversion disorder	1	1.1	2	2.1	0.9596
Apathy	1	1.1	2	2.1	0.9596
Gastaut-Geschwind Syndrome	1	1.1	0	0	
Total	95	100	95	100	

The presence of psychiatric disorders was compared before and after epilepsy surgery, with no significant differences found (OR: 1.371 (0.760-2.473), p = 0.294).

## Discussion

Complications of surgical intervention in epilepsy have been approached from several perspectives, including medical, neurological, and psychiatric complications. A large number of studies have associated epilepsy surgery with psychiatric complications and concluded that it is important to make a longitudinal psychiatric diagnosis as part of the evaluation of the potential success of surgery [6]. However, many of these studies are associated with certain methodological problems, including failure to assess the psychiatric status of patients before and after undergoing surgery, which may be explained in part by the lack of a psychiatric physician on the team in many centers where epilepsy surgery is performed [7], and the lack of training of neurologists and epileptologists in the recognition of psychiatric comorbidity should also be mentioned here. Postoperative de novo complications such as depression, anxiety, and psychosis have been reported in up to 26% of patients who underwent epilepsy surgery [5].

In the case of preoperative symptoms, their presence has been considered a predictor of poor seizure control after the procedure, although it is not considered a contraindication for epilepsy surgery [5]. Likewise, it has been observed that patients who have undergone epilepsy surgery are susceptible to psychiatric disorders in the postoperative period, either as a relapse of a controlled condition, as an exacerbation of a known process, or as de novo symptoms [7]. However, there are variable results among the different investigations, and there is a lack of studies in the Mexican population.

The psychiatric outcomes of epilepsy surgery are of great relevance given the high prevalence of psychiatric disorders in epilepsy patients. A systematic review from 2011, which included 13 articles, demonstrated that there was no improvement or significant changes in psychiatric outcomes after surgery, and only one study showed deterioration due to increased anxiety in the context of seizures that persisted after surgery [8]. The same systematic review concludes that most newly developed psychiatric disorders in these patients mainly appeared in the context of poor seizure control after surgery, surgical complications, or the need to continue medical treatment for seizures even after surgery. The findings reported in this systematic review are similar to the results obtained in our study.

A systematic review from 2013 regarding psychiatric outcomes in temporal lobe epilepsy surgery included 39 articles [9]. Depression (25 articles) and anxiety (17 articles) were the most explored disorders. Regarding depression, eight studies reported symptom improvement, one study found no significant changes, and 18 studies reported new cases, although in a minority of cases. As for anxiety, seven studies reported symptom improvement, four studies reported worsening of symptoms, and one study found no significant changes. The same systematic review mentions the surprising lack of studies on cognitive outcomes in epilepsy surgery in the scientific literature.

A study from the year 2000, which included 167 patients [4], found some remarkable findings, such as preoperative psychiatric disorders not being predictors of postoperative psychiatric disorders, patients with pre- and postoperative psychiatric disorders having a worse prognosis in terms of seizure frequency, laterality not being related to poor psychiatric prognosis, and the size of surgical resection being positively associated with the development of postoperative emotional lability.

The present study had some limitations. For example, the information was obtained retrospectively from databases and clinical records, rather than through direct interviews with the patients. Another limitation of this study was that the psychiatric diagnoses were made based on the criteria of DSM-IV and not through scales or structured interviews. Finally, we consider that another limitation was the lack of previous research on the subject in our country, partly because there are few neuroscience centers and institutions where epilepsy surgery is performed and that have the necessary multidisciplinary teams for this.

## Conclusions

In conclusion, our data suggests that psychiatric comorbidity remains without significant changes after surgery, which is consistent with findings in the literature on the subject reported in other countries. Since psychiatric comorbidity is so important and prevalent in people with epilepsy, we would also like to emphasize the importance of having patients who are candidates for epilepsy surgery evaluated longitudinally by a psychiatrist as part of the minimal investigations for this purpose.
